# Agenda setting and socially contentious policies: Ethiopia’s 2005 reform of its law on abortion

**DOI:** 10.1186/s12978-021-01255-z

**Published:** 2022-06-13

**Authors:** Sarah Jane Holcombe, Saba Kidanemariam Gebru

**Affiliations:** 1 Bill & Melinda Gates Institute for Population and Reproductive Health, Johns Hopkins Bloomberg School of Public Health, 615 N. Wolfe Street, Baltimore, MD 21205 USA; 2 Ipas, Addis Ababa, Ethiopia

**Keywords:** Ethiopia, Policy reform, Agenda setting, Abortion law

## Abstract

**Background:**

In 2005, Ethiopia took a bold step in reforming its abortion law as part of the overhaul of its Penal Code. Unsafe abortion is one of the three leading causes of maternal mortality in low-income countries; however, few countries have liberalized their laws to permit safer, legal abortion.

**Methods:**

This retrospective case study describes the actors and processes involved in Ethiopia’s reform and assesses the applicability of theories of agenda setting focused on internal versus external explanations. It draws on 54 interviews conducted in 2007 and 2012 with informants from civil society organizations, health professionals, government, international nongovernmental organizations and donors, and others familiar with the reproductive health policy context in Ethiopia as well as on government data, national policies, and media reports. The analytic methodology is within-case analysis through process tracing: using causal process observations (pieces of data that provide information about context, process, or mechanism and can contribute to causal inference) and careful description and sequencing of factors in order to describe a novel political phenomenon and evaluate potential explanatory hypotheses.

**Results:**

The analysis of key actors and policy processes indicates that the ruling party and its receptiveness to reform, the energy of civil society actors, the “open windows” offered by the vehicle of the Penal Code reform, and the momentum of reforms to improve women’s status, all facilitated liberalization of law on abortion. Results suggest that agenda setting theories focusing on national actors—rather than external causes—better explain the Ethiopian case. In addition, the stronger role for government across areas of policy work (policy specification and politics, mobilization for enactment and for implementation), and the collaborative civil society and government policy relationships working toward implementation are largely internal, unlike those predicted by theories focusing on external forces behind policy adoption.

**Conclusions:**

Ethiopia’s policymaking process can inform policy reform efforts related to abortion in other sub-Saharan Africa settings.

**Supplementary Information:**

The online version contains supplementary material available at 10.1186/s12978-021-01255-z.

## Plain language summary

Globally, deaths of women due to unsafe abortion remain high. However, few countries have changed their laws to allow safer, legal abortion. In 2005, Ethiopia reformed its law to permit women to obtain an abortion for a significantly greater number of reasons, and this reform has resulted in a real expansion of women’s access to services.

This retrospective case study uses information from interviews with 54 people involved in Ethiopia’s reform and from government and research documents to see whether explanations of the reform that focus on the roles of national actors versus on the roles of external actors and influences better explain how Ethiopia’s reform took place.

This study finds that national actors and processes were most central to Ethiopia’s reform. In particular, a ruling party open to reform, the work of the women’s movement and of reproductive health nongovernmental organizations, the ability to take advantage of political events, and the collaborative relationship between government and nongovernmental organizations all supported reform. At the time, many major external actors were either against the reform (the U.S. government) or stayed neutral.

Findings can help those seeking to understand or plan policy reform efforts in other sub-Saharan Africa countries.

**Supplementary Information:**

The online version contains supplementary material available at 10.1186/s12978-021-01255-z.

## Background

Much has been written on agenda setting and policy adoption processes in Western democratic contexts, but far less in low-income and less democratic political systems, or for socially contentious policies. Ethiopia’s 2005 liberalization of abortion law as part of the overhaul of its Penal Code provides an opportunity to explore the theories of policy adoption that are most applicable. Globally, this type of reform remains infrequent, particularly in sub-Saharan Africa (SSA), despite a general trend of liberalization [1, 2]. Like much of SSA, Ethiopia had some of the world’s most restrictive laws on abortion [3] and some of the highest maternal mortality rates, at least a third of which were due to unsafe abortion [4, 5]. Although liberalized laws on abortion are linked with reduced mortality due to unsafe abortion [6, 7], at the same time, the associated legal reform processes reliably rouse public conflict and often do not increase stakeholder support [8, 9]. Conflict appears to be an even more obvious outcome in more traditional societies [10, 11] such as Ethiopia, where over 63% of the population views abortion as “never justifiable” [12].

This paper’s objective, using a retrospective case study approach and process tracing, is to describe the abortion law liberalization process in Ethiopia between 1991 and 2005 and assess the applicability of theories of agenda setting. We identify the actors and mechanisms at work, highlight features of the political environment and structure (the political opportunity structure) that facilitated or inhibited the rise of this issue to the national political agenda, and contrast the explanatory value of policy theories emphasizing national forces (agenda-setting theory) or external forces (international policy diffusion).

This study starts with the hypothesis that the key forces behind national reform of abortion law in Ethiopia are homegrown. Our contention is not that international influences are absent, but that we should look first at national factors when the policy involved is culturally sensitive and where financial or other geostrategic political pressures appear less relevant. We argue here that the key factors are at the national level, and that they include government leadership, national nongovernmental organization (NGO) contributions (particularly from women’s rights NGOs and a medical professional society), a collaborative rather than a conflictual civil society-government relationship, and the opportunities offered by the Penal Code reform process and by the momentum of advocacy efforts to empower women and improve their socioeconomic status. Broad international background influences, such as foreign financing of the health sector or foreign donor policies, were present but operated in ambiguous or countervailing directions. Some smaller donors broadly supported organizations active in the sector, but Ethiopia’s primary donor engaged in significant, explicit opposition to reform. Accordingly, we argue that theories of agenda setting that highlight a primary role for national actors—particularly government in shaping policy selection and politics, and national civil society organizations—map better to how Ethiopia’s reform took place. The examination of politics here focuses on the deliberate activities undertaken by stakeholders to secure outcomes through government.

Abortion law liberalization is a notable outcome for both normative and theoretical reasons. At the individual level, it is a policy reform central to women’s right to self-determination and agency over their bodies with the potential to prevent avoidable death and disability for women. At a societal level, policy debates over abortion often reveal deep social and political cleavages on women’s roles, sexuality, and religious values [13] and in the West are often battles by civil society actors to gain government enforcement of a particular world view [9]. Because of its personal and cultural salience, policy on abortion often sparks significant elite and mass public political engagement in affluent democratic countries [8, 14]. Finally, Ethiopia’s reform stands out globally because it has resulted in real increases in women’s actual access to and use of safe, legal abortion services rather than remaining an aspirational policy. Next only to South Africa, Ethiopia arguably now has one of the most progressive laws in practice in sub-Saharan Africa [15]. This inspires questions about what theories of agenda setting best explain Ethiopia’s successful reform. The relative infrequency of impactful reforms such as Ethiopia’s led us to select it for study.

Ethiopia is a nation of more than 100 million people located in the horn of Africa; the urban and educated are but a narrow sliver of its overwhelmingly rural population [16]. It is one of the world’s poorest countries, ranking 169th of 177, with an annual per capita income of $700 [17]. Ethiopia is also a highly religious and traditional society where all religious groups (Ethiopian Orthodox Christians, Muslims, Evangelicals, and followers of traditional beliefs) are officially opposed to abortion except to save the life of the woman [18].

Ethiopia has had limited experience with democracy, elections, and NGOs. Until 1974, Ethiopia was a largely feudal monarchy, and the subsequent 17 years under the Derg regime were repressive and violent, leaving little room for the emergence of civil society or any reform of the country’s laws. After coming to power in 1991, the new ruling party (Ethiopian People’s Revolutionary Democratic Front, or EPRDF), internally led by members of the Tigray People’s Liberation Front (TPLF), brought order, a “developmentalist” agenda, economic growth [19, 20], emergence of a free press, and a blossoming of civil society up until a deadly post-election crackdown in 2005 [21, 22]. Reform of Ethiopia’s abortion law took place during this unprecedented space for political expression and civil society growth and activity. The country’s current political structure consists of a federation of ethnically based regions, of which the three largest contain more than 80% of the country’s population [23–25].

Ethiopia has a thin and emergent NGO sector. Only after the arrival of the current regime and the return of Western donors did the numbers of NGOs mushroom from fewer than 60 (most of them international) at the end of the 1980s to almost 2000 in 2007 [26, 27]. Only after the EPRDF took power in 1991 were the key professional societies and NGOs involved in Ethiopia’s reform established and did related international NGOs arrive. Similarly, Ethiopia’s educated and political elites have historically been extremely small, with a tremendous education and income chasm between them and the rest of the country [28].

Ethiopia’s 1957 law only permitted abortion in case of “grave and imminent danger to the life of the woman” and required approval from two physicians [29]—an especially high bar, given the country’s chronic shortages of physicians and their near total absence in rural Ethiopia where 87% of the population lives [30]. Ethiopia’s reform—the law and its 2006 regulation—significantly increased women’s access to abortion services. First, the permissible circumstances for terminating a pregnancy expanded to include cases in which there is rape, incest, or fetal impairment; pregnancy continuation or birth endangers the health or life of the woman or fetus; or the woman has physical or mental disabilities or is a minor (under 18) who is physically or mentally unprepared for childbirth [31]. The law also recognized women’s testimony as sufficient to determine eligibility for services in cases of rape or incest. The Ministry of Health’s subsequent Technical Guidance further expanded access. It permitted minors to access services without requiring documentation or parental, legal, or medical approval; authorized new categories of clinicians (midwives, nurses, health officers) to offer abortion services and mandated training; enabled service provision in public and private facilities of all levels; and required that services be provided within three working days [32, 33].

Ethiopia’s reform is one of the few globally that has markedly expanded access to care. Many legal reforms languish unimplemented or poorly implemented [6]. In Ethiopia, public sector statistics show that women are accessing services, that maternal mortality due to unsafe abortion has decreased, and that increased proportions of women are adopting contraceptive methods post abortion [4, 34–36].

### Candidate theoretical explanations

Broadly, two candidate sets of theoretical frameworks to describe the actors and processes are that of agenda setting, developed in the U.S. context [37, 38] and focusing exclusively on national actors, and frameworks pointing to external actors or forces such as World Polity Theory or the international diffusion of norms.

Kingdon’s theory of policy adoption is a paradigmatic example of a theory focusing on national actors. It posits three interacting streams of internal processes shaping national governmental agenda setting—problems, policies and politics [38]. Within these three streams, civil society actors identify problems and specify policy solutions and governmental actors select among alternatives and make formal decisions. Actual policy adoption occurs only when policy entrepreneurs, from in or outside government, capitalize on opportune moments (“open windows”) and connect a problem to a policy. They then shepherd the policy proposal through political processes. Related insights from study of sub-Saharan African policymaking point to a larger role for the state, the influence of elite groups, and a more collaborative civil society–state relationship [37, 39–41].

Alternative theoretical frameworks instead emphasizes external forces, including international diffusion of norms that fueling the converging resemblance of national policies [42, 43], with international organizations, networks of experts, and NGOs as key mechanisms for this transfer [44, 45]. Variants additionally point to more coercive mechanisms (states exerting financial coercion, violence, etc.) [46]. This study seeks to assess applicability of the first set of more internally focused theories.

## Methods

Our retrospective case study of abortion law reform in Ethiopia has the goal of supporting causal inference by identifying key factors and the possible routes by which they affect the outcome of abortion law reform [47]. We draw on three main data sources: interviews; government policies, plans and evaluations, donor agency and grantee reports, and newspaper articles; and published secondary research on abortion policy adoption processes. Our primary data source is in-depth interviews conducted in 2012 (7 years post reform) with a purposive sample of 54 individuals knowledgeable about the reform of the Penal Code with respect to abortion law, who were selected using reputational and positional criteria. Interview guide questions asked about the reform sequence and chief actors, the framing of the debate, the reform roles and actions of the informant’s organization, and summative questions on the factors informants saw as most central to explaining Ethiopia’s 2005 reform. Ethical approval was obtained from Addis Ababa University and the University of California, Berkeley Committee for Protection of Human Subjects.

Our analytic methodology is within-case analysis using “process tracing,” an approach using pieces of sequential evidence from multiple data types from within a case (“causal process observations”) to identify the mechanisms or processes through which a factor affects an outcome. These causal process observations are the data or “clues” that aid in assessing the relative strength of the relationship between actors and forces and the outcome of reform [48, 49]. They emerge from interviews but also from newspaper and secondary data sources. Process tracing enables an assessment of the implications of evidence for the researchers’ hypothesis of interest (and for alternative hypotheses) through use of tests of necessity and/or sufficiency.

After completing data collection and coding, we reviewed interview transcripts and notes; government, donor, and NGO documents; and press coverage in order to cross-check statements made by interviewees and to fill any gaps. Project reports and press coverage during the reform period helped more precisely date events and evaluate the accuracy of interviewee statements about the importance of their involvement or the types of reforms for which they called. We then used the data to produce a chronology of the reform process, to identify key leaders and events, and to develop propositions about which factors were most linked with the outcome of reform (Additional file 1).

## Results

We conducted interviews in English in Addis Ababa, Ethiopia, in February and March 2012. We also used another investigator’s 2007 Amharic-language interviews with one religious leader from the Ethiopian Orthodox Church (EOC) and one from the Supreme Council of Islamic Affairs (SCIA), conducted in Amharic and summarized in English [50]. All individuals invited agreed to be interviewed. Men were 60% of all informants, and ages ranged from mid-30 s to 70 s. All were professionals and senior figures in their institutions; all but two were currently employed and were Ethiopian nationals. Unless noted, all quotations are from Ethiopian informants (Table 1).

**Table 1:** Interviewee background (2012 and 2007)

Government	6
Women’s rights NGOs	4
Ethiopian (reproductive) health NGOs	9
Reproductive health medical professionals	10
Researchers	6
International nongovernmental organizations	12
Media	1
Donors	4
Religious leaders (Ethiopian Orthodox Church, Supreme Council of Islamic Affairs)	2

Primary affiliation only

Interviews lasted between 20 minutes and four hours, and all but ten were recorded and transcribed verbatim. Unrecorded interviews were summarized shortly after the interview concluded. All interview transcripts or write-ups were then coded using HyperResearch software [51]. Transcripts were coded to identify actors, interests, roles, and sequence of key events.

### Reform chronology

Although maternal mortality from unsafe abortion was high during the 1980s in Ethiopia, the previous regime, the Marxist Derg government, enacted no related reforms. The 1991 advent of a new political regime led by the EPRDF, a coalition of forces headed by the TPLF, brought a government receptive to reforms to improve women’s status and initially opened political space for civil society to emerge and act. The swift adoption of progressive national health, population, and women’s policies in 1993, and particularly the new constitution in 1995, made it clear there would be a complete overhaul of the 1957 Penal Code, with an explicit eye for expanding the “democratic rights and freedoms” of women [52].

Starting in the mid-1990s, newly founded civil society organizations began advocating for a broad set of reforms to improve women’s legal status, including on abortion—most notably, the Ethiopian Women Lawyers’ Association (EWLA). The Ethiopian Society of Obstetrician-Gynecologists (ESOG) involvement began in the late 1990s and focused more narrowly on reproductive health and on Penal Code provisions related to abortion and contraception. A number of other leaders from the growing community women’s organizations, Ethiopian reproductive health NGOs, and health professional groups also advocated for more progressive policies on abortion. Ipas, an international NGO, played a central role. In 2003, these and other organizations joined to coordinate their work through the Advocacy Working Group (AWG), a core set of NGOs committed to liberalizing the country’s laws on abortion. The AWG also voted to have the unofficial but regular participation from the head of the National Office of Population, which as a government body could not participate publicly.

Throughout this period (2001–2009), the United States, under the leadership of the Bush administration, reinstituted the Mexico City policy (known by opponents as the “Global Gag Rule”) and made receipt of assistance for non-U.S. NGOs conditional on abstaining from work related in any way to abortion [53, 54].

In 2000, the Ethiopian Parliament launched overhaul of the Penal Code by requesting a draft from the executive branch and received two different versions later that year from the Ministry of Justice and the Federal Institute of Law Reform. After a full hearing on the drafts, Parliament sent sections related to women and reproductive health to three standing committees (the Women’s, Social, and Legal Affairs Committees), the last charged with shepherding the sections through review and passage. NGO reform supporters were prompt to air concerns about the lack of improvement in these first drafts. EWLA publicly noted the absence of any change to the 1957 language on abortion as well as the failure to include a number of other measures to improve women’s status, such as language prohibiting domestic violence and female genital cutting/mutilation [55, 56]. The AWG reached out to parliamentarians to offer technical and other background support. Parliamentarians then tasked the AWG with carrying out formal public outreach on the elements of the revised draft of the Penal Code related to women, reproductive health, and abortion and gathering public opinion. AWG members presented at hearings on the draft Penal Code in 16 cities in order to build broader understanding and support for the reforms among the public [57]. During the peak of reform activities between 2001 and 2003, EWLA, ESOG, and other leaders also presented in public forums, including on television, on the radio, and in Parliament [58, 59]. At this time, the AWG and its members were one of many groups and individuals working with parliamentarians to revise and update the Penal Code.

After the regional hearings, the full Parliament held 5 days of hearings in 2003 on all elements of the Penal Code reform related to women. AWG members helped the Legal Affairs Committee design the hearing agenda, and reform supporters provided presentations touching on abortion. Presenters were obstetricians-gynecologists (ESOG) and lawyers (EWLA) but were also sociologists and other social science researchers, and there were three commissioned research papers on abortion. At this point in early 2003, the proposed Penal Code language circulating in Parliament would have completely decriminalized abortion [57].

However, at this late stage, formal opposition surfaced. First, two small public demonstrations of anti-abortion physicians occurred in the main public space of the capital city, Meskel Square, organized by the Christian Workers Union for Health Care in Ethiopia [57, 59]. These protests were notable not for their size but for the fact that they occurred at all. Public demonstrations opposing proposed government policies in Ethiopia were rare to nonexistent in this period. Also at this time, anti-abortion print and film materials began circulating in Addis, particularly in Protestant Evangelical communities and at some religious services [60]. The most forceful opposition came in December, when the Patriarch of the Ethiopian Orthodox Church, the leader of Ethiopia’s historically dominant religious group, publicly denounced abortion as a sin and stated that reform was unacceptable. He sent a press release to the capital’s leading newspapers with a statement and communicated directly to the President, Prime Minister, and Parliament (House of People’s Representatives) voicing opposition [61]. The Patriarch did not attack government but instead the civil society groups proposing reform. The Roman Catholic Church, a far smaller group whose members make up only about 1% of Ethiopia’s population, also sent the government a communication opposing reform.

Government promptly responded to this opposition. The Legal Affairs Committee recommended that AWG members put advocacy work on hold and keep a low profile. The committee then held a hearing for the full Parliament where reform opponents presented *The Silent Scream*, a film that has been a staple of U.S. anti-abortion advocacy work [62]. For the next year, the reform dropped out of public sight. The final version of the Penal Code approved in May 2004, issued in June 2005, did not include the complete decriminalization of abortion law previously proposed. Instead, the Legal Affairs Committee, in consultation with the AWG, included a lengthy list of legal exceptions to criminalized abortion. The final legal language still permitted significantly increased access to legal abortion services, though in a less transparent manner. Reform supporters explained the law as a compromise worded conservatively to mollify opponents. Further, Parliament delegated development of technical guidelines to the Ministry of Health, who later relied on reform-minded technical experts to craft these guidelines grounded in global (World Health Organization, or WHO) evidence-based standards. These 2006 guidelines minimize hurdles and maximize women’s actual access to services [32]. Table 2 provides a chronology of the overall reform and allied events.

**Table 2:** Timeline of Ethiopia's reform of its Penal Code with respect to abortion

**Ethiopian government**	EPRDF government comes to power		National policies on Population, Women, and Health issued		Enactment of Federal Constitution (Articles 25, 35 on women’s rights)				Women’s Affairs Standing Committee of the House holds workshop on Penal Code	•Parliamentarians direct Ministry of Justice (MOJ) to propose revisions to 1957 Penal Code •Revised Family Code goes into effect •Release of two executive branch drafts of new Penal Code with no change related to abortion		•Two draft Penal Code reform versions from MOJ & FILR (Federal Institute of Legal Reform) sent to Parliament •FILR releases amended draft Penal Code proposing decriminalization of abortion •Government—NGO AdvocacyWorking Group (AWG) convened •Regional meetings on Penal Code reform with AWG contributions	•Senior executive branch policy committee votes to decriminalize abortion •Parliament directs Legal Affairs committee to seek input from public and experts and reconcile executive branch Penal Code drafts •Week-long workshop for full Parliament on RH and HTP issues in draft Penal Code	•“Silent Scream” screened for full Parliament •Revised Penal Code approved without debate	•New Penal Code goes into effect •National elections—crackdown on opposition	MOH Technical Guidance on Abortion issued			Enforcement begins of Civil Society Organizations Law restricting NGO advocacy
**Ethiopian civil society**		•Ethiopian Society of Obstetricians & Gynecologists (ESOG) founded •Ethiopian Midwives Association (EMwA) founded		ESOG 2nd Annual Meeting topic: unsafe abortion	Ethiopian Women Lawyers Association (EWLA) founded				ESOG 7th Annual Meeting topic: "Illegal and Unsafe Abortion"	EWLA workshops held on FGM and development of Penal Code reform recommendations	Televised symposium on unsafe abortion ESOG, WALTA Information Center	•ESOG research and reform recommendations released •ESOG President publicly calls for liberalization of the abortion law	•Anti-abortion demonstrations in Addis •EOC Patriarch condemns any liberalization of the law						
**National/ International**				Ethiopian President opens International Conference on Population and Development (ICPD)	Kenyan ob-gyn presents to ESOG on abortion law reform				Ramp up of Packard Foundation funding for reproductive health	Ipas establishes office in Addis	Family Guidance Association of Ethiopia refuses to sign GGR, is barred from receiving US funding								
**International activities and actors**	International Safe Motherhood conference in nearby Nairobi			International Conference on Population and Development (ICPD) in Cairo	Fourth International Conference on Women (Beijing)					U.S. Mexico City Policy (Global Gag Rule or GGR) reinstituted, barring support to non-US NGOs in any way involved with abortion									U.S. Mexico City Policy/ GGR rescinded
	1991	1992	**1993**	**1994**	**1995**	**1996**	**1997**	**1998**	**1999**	**2000**	**2001**	**2002**	**2003**	**2004**	**2005**	**2006**	**2007**	**2008**	**2009**

## Analysis and discussion

### Roles of national and international actors and conformance with theory

Ethiopian ruling party leadership and leaders of key committees in the Parliament played central roles in reform. The government’s well-known progressive and secular orientation, its track record of progressive policies both before and after coming to power in 1991, and the allied democratic breathing space it left for NGOs prior to 2005, all served as encouraging precedents for later national reform of the overall Penal Code and the abortion law. Additionally, the government’s characteristic resistance to foreign pressure and its channeling of civil society contributions to meet national goals, further smoothed the path of reform [63, 64].

The ruling party’s past reforms to improve women’s status and its receptiveness to further reform emboldened civil society reform supporters. The party was known to be secular, had enacted several substantial policy reforms to improve the status of women, and prioritized improving women’s status as a necessary step toward achieving socioeconomic development [65–67]. Interviewees saw the ruling party’s long-standing political ideology and experience as predisposing it to further progressive reforms related to women and reproductive health.

The government has a good and strong position on promoting women’s rights, starting right from the bush. There were many women soldiers involved in the fighting.—*Interview 44* (Obstetrician-gynecologist)

Reform supporters leveraged several supportive national legal precedents—most centrally, the new constitution. Ethiopia’s 1995 adoption of a new constitution meant that the Penal Code would need substantial revision, setting in motion later reform processes into which even a controversial reform (such as that of abortion law) might be fit. The constitution’s preamble directly calls for rectifying historical injustices and elimination of discrimination and later calls out the need to address historical injustices against women [52]. Reform supporters, particularly women’s rights activists, explicitly used the constitution as a precedent in their arguments for reform.

The constitution is a mother to everything. The Penal Code is an answer to the constitution. The Women’s Policy focuses on the right of women not to be hurt, the right of women to work, participate. All policies are intertwined. If there had been no Penal Code reform process, there would not necessarily have been any reform with respect to abortion. —*Interview 36* (Researcher)

The Ethiopian government had consistently demonstrated willingness to ignore or to counter opposition, whether from foreign donors or religious groups [19, 25, 68, 69]. For example, the Prime Minister at the time complained to the U.S. ambassador about Rep. Chris Smith’s interference in Ethiopian domestic politics over abortion during the Penal Code reform [70]. The ruling party up to this point had also historically been undeterred by religious opposition, including from the Ethiopian Orthodox Church [71].^1^ This ability to resist direct internal religious demands as well as U.S. pressures suggests that Ethiopian government leaders would be unlikely to be swayed by more diffuse external forces.

Further, although interviewees described instinctive government resistance to foreign pressure, they noted that international organizations could make contributions as long as they were aligned with overall national policy.

In the case of Ethiopia, especially the government, they don’t much like interference and the pushing [from] outside. In fact, when you try to push and impose, they just go against that. So, I don’t think any push from an international organization would result in anything. But the role has to be…seen as complementary, and it has to be within the policy of the government. For example, if you take the issue of abortion or any other provision of the criminal court, if it was Ipas pushing that, then I don’t think it would be [effective].—*Interview 37* (Women’s rights leader)

The period before the 2005 elections offered special space for NGO advocacy and collaboration with government. The growing numbers of active civil society organizations, a relatively free press, and government receptiveness to structured civil society involvement in Penal Code reform enabled those in the reproductive health field and women’s rights advocates to contribute. Interviewees described the government’s model of national policy change as one in which education and outreach to build public support for policy enactment were viewed as prerequisites and keys to actual policy implementation—a modus operandi dating back to its roots as an insurgency movement [65, 72].

However, NGO informants saw government receptiveness and support as a prerequisite for, but not a guarantee of, reform. They pointed to an initiating role for civil society reform supporters, who had to bring the proposed policy to government leaders and also to educate the public and generate support.

Ok, you bring it forward, the [government] is not going to cook it and finish everything and give it to you. …You need initiators to take it up to the top of the ladder, to make sure it'll be blessed by them. It’s progressive, they have been for it, and they were for it and they encouraged the movement to take its course, they facilitated everything, took it up to the Parliament and had it passed through. —*Interview 33* (Obstetrician-gynecologist)

Interviewees understood NGO public advocacy as central to initiating and to keeping reform efforts on the radar screen and described civil society as a key source of policy options and ideas.

The catalyzing role is still the role of civil society organizations. The government has a political commitment, but they also have different priorities. They have priorities to focus on, and sometimes they are not even sure of how to combine, and what is the right policy and what is the right sequence…. So there comes [a role for], especially, research-based, knowledge-based organizations. There are lots of gaps even now in the law. —*Interview 37* (Women’s rights leader)

Consistent with Kingdon, NGOs were the source of many of the policy ideas that expanded access to services [38]. For example, after the 2004 anti-abortion opposition surfaced, the AWG helped supply language that the Parliamentary Committee used to replace decriminalization in the final version of the Penal Code.

Starting in the mid-1990s, several civil society organizations began advocating for a broad set of reforms to improve women’s legal status, most notably, the EWLA. Founded in 1995. EWLA pursued a comprehensive agenda to improve women’s socioeconomic status and situated abortion law reform among this broader set of needed reforms, seeing them all as inescapably linked. To equip itself to contribute to Penal Code reform, EWLA documented Ethiopian women’s adverse cultural and legal status and analyzed applicable Ethiopian and international laws [55, 73, 74]. In 2000, it presented recommendations to federal and regional authorities on criminalization of domestic violence, liberalization of the abortion law, and the repeal of legal amnesty to abductors and rapists who marry their victims. EWLA representatives spoke publicly about “women’s right to life,” the injustice of maternal mortality due to unsafe abortion, and the need to revise and liberalize the law on abortion [55, 75].

A striking feature of EWLA’s advocacy, and that of other groups involved in the AWG, was the often close and amicable connections between civil society and government leaders. An emblematic example occurred during a television interview, when EWLA’s leader posed a question to the Prime Minister about abortion: “Would you support the amendment of the Criminal Law [Penal Code] in terms of relaxing the provision of the law and making it more flexible to benefit more women?” In his response, the Prime Minister not only signaled his tacit support for reform but also his expectation that those outside government should mobilize and demonstrate public support for reform.

Ethiopian obstetricians–gynecologists (ob–gyns) and their professional association (ESOG) were the next most visible nongovernmental group active on reform of the Penal Code law on abortion. Because ob-gyns are well-educated, more affluent, and generally male, they bring special social capital to public debates [76, 77]. They are also regularly called on to contribute to government reproductive health policy development, program design, and training. ESOG was founded in 1992 with an explicit mission to address Ethiopia’s elevated maternal mortality. Ob-gyns and ESOG were central to identifying maternal mortality due to unsafe abortion as a problem requiring policy attention; they framed the need for reform as one of public health [78]. Even before ESOG’s founding, individual ob-gyns produced much of the core research on the topic; brought evidence of the scope of maternal mortality, including that due to unsafe abortion, to the Ministry of Health; and urged policy action [5]. ESOG leadership later also made a series of public calls for reforming national abortion policy.

However, ESOG and other reproductive health groups were active at a cost. By working on reforming Ethiopia’s law on abortion during the 2001–2009 period, despite the U.S. government Mexico City policy (the Global Gag Rule), ESOG leaders and other national reproductive health groups knew that they were forgoing U.S. government support [78]. Several NGO leaders contributed to reform as individuals acting in their personal capacity, while other NGOs decided outright to forgo funding. The Family Guidance Association of Ethiopia and Marie Stopes International, Ethiopia (MSIE) decided to provide safe, legal abortions and forfeit U.S. assistance. Ethiopia is notable for being one of the very few countries where this occurred [79, 80].

Ethiopia’s reform is conspicuous for the absence of formal active intervention to oppose reform from either domestic or international sources until quite late in the process. Most studies, whether they be of abortion policy specifically [81] or of morality policy more generally [9, 82–88], emphasize the role of religiously motivated opposition to abortion law reform. However, there was limited public expression of local anti-abortion opposition (a 2003 doctors’ demonstration with support from U.S. anti-abortion groups, then statements from the Ethiopian Orthodox Church). While this opposition did trigger a delay and wording change in the Penal Code language, it did not alter the actual direction and consequences of the reform [62, 89].

####  International norms, actors, and influences

Ethiopia’s heavy reliance on foreign assistance to finance government spending (particularly for the health sector), the in-country presence of donors and the host of international NGOs they support, and Western tertiary education of many in Ethiopia’s leadership, all suggest the need to examine international influences on national policymaking. However, the ambivalent nature of international norms related to abortion, along with U.S. government anti-abortion policies aimed at deterring reform, suggest that external support did not weigh heavily in enabling Ethiopia’s reform.

If it existed, international consensus among countries, international organizations and NGOs about abortion and abortion law might well influence country-level policy deliberations. However, endless U.S. partisan brawls, growing European Union conflicts [82], and the recurrent and increasing conflict between supporters and opponents of sexual and reproductive rights in United Nations (UN) venues, coupled with traditional UN system reluctance to push legal change on member states [90, 91], show that international consensus on abortion policy (let alone liberalization of abortion laws) has been weak to absent. Although there may be limited agreement that abortion should be legally available for a narrow set of conditions (e.g., rape, incest, threat to the life of the woman), individuals and groups operating at national and international levels actively contest even many of these grounds [91–93].

At the same time, two factors support the idea that there is an emergent international norm of support for liberalized abortion laws. Pivotal international conferences focusing on reproductive health, such as the 1994 International Conference on Population and Development where Ethiopians played a key role, have endorsed a focus on the needs and rights of individual women. Further, UN and other international meetings and statements have increasingly endorsed the idea that abortion services should be available where legal [94]. However, Catholic and international evangelical Christian organizations are increasingly active in countering these norms in global meetings and national settings.

International agencies have had strong incentives to stay on the sidelines of efforts to reform national law on abortion. Although several (the United Nations Population Fund [UNFPA], WHO) have been players in the development of the reproductive health field globally [95], they have a base level reluctance to push member states to undertake legal reforms generally, particularly regarding sensitive topics such as abortion [92, 96]. They have also sought to avoid political and financial repercussions from the United States under Republican administrations. For example, the United States has repeatedly withheld funding due to alleged UNFPA support for abortion services [90, 91, 97, 98].

Bilateral donors have either given limited support to or, in the case of the United States, have actively opposed abortion law reform. Up through Ethiopia’s 2005 reform, few bilateral donors directly supported services related to abortion, with limited exceptions from the Netherlands, Sweden and Norway. Their funding tended to be for women’s rights more generally or for post-abortion care [99–101]. Because of either their own opposition to reform or their recognition of recipient country autonomy and the sensitivity of the issue, up until recently, donor countries have typically not promoted liberalization of abortion laws, even indirectly. U.S. foundations supporting organizations in Ethiopia are prohibited by U.S. law from funding work to influence legislation [102], although several have given support to national and international organizations working on post-abortion care training and delivery and to public education and advocacy related to reproductive health services (including abortion).

In fact, U.S. government policy (the Mexico City policy/Global Gag Rule) in place during the Penal Code reform explicitly aimed to discourage abortion law liberalization. U.S. opposition was significant, due both to the amount of U.S. support to Ethiopia and to how it was channeled. U.S. foreign assistance to Ethiopia, particularly for the health and reproductive health sectors, dwarfs that of other donors [103, 104], and almost 80% of U.S. health assistance is channeled through NGOs, both international and Ethiopian [70]. Thus, NGOs had a powerful incentive to avoid participating in reform efforts and jeopardizing their U.S. funding.

However, U.S. government opposition to reform had multiple and countervailing effects. Although formal U.S. policy was intended to discourage legal reform, U.S. historical support for the reproductive health sector had arguably strengthened the presence of potential reform advocates in Ethiopia. Further, supporting abortion law reform became a way to stand up against a powerful country seeking to push others into following its policy advice in a culturally sensitive topic area. The overt U.S. government opposition to reform also made it harder for Ethiopian reform opponents to discredit supporters as foreign inspired and Western.

During this period in Ethiopia, a few international NGOs focused on provision of abortion services or related national-level advocacy, with work primarily on training and service delivery [91]—primarily Marie Stopes International Ethiopia, headquartered in the United Kingdom, and Ipas from the United States. MSIE provided comprehensive reproductive health services including abortion in Ethiopia but did not engage in advocacy in order to avoid jeopardizing its service delivery work. Pathfinder International, a U.S. NGO, was supportive of legally expanding access to safe abortion but, as the main cooperating agency for the United States Agency for International Development in Ethiopia, did not participate due to the Mexico City policy.

Of the NGOs, Ipas was the most engaged and played a special boundary-spanning role. Although an international organization, Ipas hired a local director experienced in aligning organizational with national objectives, and added training, work on contraception, and increased engagement with policy. During reform, Ipas supported policy-related research, backstopped the Advocacy Working Group, and shared relevant resources and experience from other settings. Table 3 summarizes the valence or direction of actors and other forces influencing reform.

#### The political opportunity structure and mood

Consistent with Kingdon, national reform supporters capitalized on at least two key opportunities (open windows) that heightened the chances of reform [38]. First, the Penal Code reform provided both a vehicle to include a liberalized law on abortion as well as space for civil society activism. Second, the momentum of a larger movement to improve women’s status provided added impetus for abortion law reform.

First, the overhaul of the country’s Penal Code was key to the liberalization of abortion law. The violence and instability of the previous Derg regime had prevented Penal Code reform for 17 years. After 1991, the adoption of the new constitution made it clear that the entire Penal Code would have to be updated, and thus there was opportunity available for reform of the law on abortion.

The country was in a process of aligning the Penal Code with the new constitution. There was a whole number of issues included in the reform, including harmful traditional practices, sexual violence, abortion…. It was an opportunity that was used wisely. —*Interview 22* (obstetrician-gynecologist)

Reformers concluded that it would be far easier to include reform within the overall legal reform process initiated by government rather than to single out abortion law for scrutiny. This less visible approach helped avoid rousing active opposition from traditional leaders, leaders at the regional levels, some members of Parliament (some of whom were EOC priests), and even the elite public [105].

Further, the Penal Code process opened up room for civil society contributions. The Ethiopian government had the expectation that NGOs and individuals would contribute their expertise to updating the country’s Penal Code. One informant commented that reform supporters had the “feeling that there is an opening, that if we push we’re not going to be punished” (*Interview 38*, international NGO leader).

Second, the momentum behind reforms to improve women’s overall status also facilitated reform on abortion law. EWLA, allies, and government were committed to improving women’s socioeconomic well-being and legal status and began work as soon as the government came to power in 1991. EWLA pursued a broad agenda of reforms, of which abortion law liberalization was one [55, 106].

So, the rape issue, the violence issue, the (harmful) traditional practices…. Abortion could be like for many a minor issue, for many. But if you see the whole issue of violence against women, especially abduction, rape, all of these…, most people could agree. Mostly. Abortion, not as much—but still, if you have the broader issues and include them within, you don’t have to bring up every issue and try to get consensus.—*Interview 6* (International NGO leader)

EWLA saw an intrinsic logic and also a strategic advantage to addressing abortion law reform within a larger package of reforms redressing historical and cultural wrongs to women.

Respondents also emphasized how what Kingdon describes as the political mood was favorable for policy reform. In 1991, Ethiopia left behind the grim rule of the Derg regime: the massacres of the Red Terror, political instability, famine, and war. A more open and optimistic spirit prevailed, along with expectations of progressive reform. Educated Ethiopians recognized that existing laws were long overdue for reform. Women’s advocates felt that reform was not only possible but was also promoted by the government. This spirit of possibility accompanied the country’s moment of democratic openness prior to 2005.

Given Ethiopia’s cultural conservatism, (re)framing the issue of abortion was seen as key to successful reform [75]. ESOG members were central to publicly framing abortion law reform as a critical public health response to the country’s extraordinarily high rates of maternal mortality. AWG members saw it as more effective to discuss reform as a way to reduce high maternal mortality and thus reach more conservative people.

It was never about rights in Ethiopia. It was about preventable deaths and that women are not only dying, but also suffering. That women and families have been going through illicit abortion. …I mean, most hospital beds were occupied by septic abortions and women were dying right and left like flies. —*Interview 26* (physician, NGO*)*

Women’s rights activists were equally thoughtful about choosing the most effective communications strategy for Ethiopia’s socially conservative context. They also did not frame access to abortion as a matter of rights, but instead talked about women dying (“women’s right to life”) and public health measures to make it harder to deny the necessity of reform.

Especially in traditional countries like Ethiopia where no aspects of women’s rights can be taken for granted, approaching the right to abortion from the public health perspective of preventing deaths and injuries from unsafe abortion, rather than from a rights-based perspective, may appeal to a broader constituency. [75] —(EWLA Executive Director)

Table 3 summarizes the valence or direction of the influence of factors linked with reform. This research has limitations, primarily recall and social desirability bias, as does all retrospective research. A series of interviewing strategies helped manage social desirability bias: knowing the topic area, interviewing a wide variety of informants, asking about the roles of other actors, asking informants to critique their own assessments, saving questions about impact for a later stage, and knowing the likely direction of informants’ biases [107–109]. Further, the social stigma associated with abortion may have decreased likelihood of subjects making exaggerated claims about their contributions to reform, as may interviewees’ awareness that a range of actors involved in reform were being interviewed. Recall bias is likely less of an issue because the reform was recent, and most interview participants were deeply involved with it. Cross-checks with other interviewees and newspaper reports also enabled us to assess the bias in interviewees’ statements.

**Table 3:** National and external factors supporting or countering reform

	National	External
*Actors*		
Supportive government (ideology, precedents) disposed to resist external interference	Supporting	
Active women's rights movement	Supporting	
Mission-driven medical profession	Supporting	
Ethiopian Orthodox Church and evangelical opposition	Countering	
International NGO supporting reform		Supporting
Absence of global consensus on abortion law liberalization		Ambiguous
U.S. government opposition to reform (Mexico City policy)		Countering
*Political opportunity structure*		
“Open window”: opportunity of Penal Code reform (allied historical moment of democratization)	Supporting	
“Open window”: successful momentum behind a broad agenda for improving women's status and well-being	Supporting	
Pent-up popular expectation of policy and legal reform after overthrow of the Derg regime (“mood”)	Supporting	
Religiously conservative population	Countering	
*Strategies*		
Abortion law reform as part of a package of reforms to improve women's status	Supporting	
Frame used: maternal mortality prevention/public health promotion—“women’s right to life”	Supporting	
*Material conditions*		
High maternal mortality due to unsafe abortion and related research base	Supporting	

## Conclusion

This analysis has sought both to identify key actors and processes present during Ethiopia’s enactment of a socially contentious policy (abortion law liberalization) and also to assess the applicability of theories of policy adoption from affluent democratic contexts. It identified national actors and processes as those most prominent in Ethiopia’s 2005 Penal Code reform. Most notably, reproductive health and women’s rights NGOs, the then ruling party and its receptiveness to reform, and the “open windows” offered by the vehicle of the Penal Code reform and the momentum of reforms to improve women’s status all facilitated Ethiopia’s liberalization of its law on abortion. The final reform, despite compromise in the face of last-minute religious objections, was still broad enough to successfully enable women’s access to services and advance rights. International actors and forces operated either in opposition to reform or in indirectly supportive ways.

Ethiopia’s civil society identified, framed, and elevated reform as the response to the problem of maternal mortality due to unsafe abortion. Framing the reform as a strategy to address maternal mortality was considered more productive than a strategy of explicit support for women’s rights. Taking advantage of a democratic opening during the time of Penal Code reform, Ethiopian reproductive health and women’s rights groups suggested policy alternatives (approaches to liberalizing the law on abortion) for government consideration. Civil society leaders and experts productively capitalized on the opportunity of the Penal Code reform, and government actors readily made use of their contributions.

This Ethiopian case also shows both the existence and utility of a more collaborative relationship between civil society and government than would be predicted by theorists. Senior government leadership welcomed including an even more progressive version (decriminalization) of the reform than was initially proposed by civil society in its overall project of updating the entire Penal Code. Leaders saw the move as in keeping with their historical commitments and policies to improve women’s socioeconomic status. This collaborative partnership between government and civil society would later prove helpful in developing the regulations to implement the new law.

The government’s understanding of women’s well-being as a prerequisite to national development, and of the need for access to safe, legal abortion in order to prevent maternal mortality, were central to its support for reform. Those seeking to enact similar policy reforms in SSA could profitably explore using these frames in their settings. Reformers in Colombia and Uruguay have used public health framing. Nonetheless, the apparent effectiveness of public health arguments does not mean rights-based arguments should be ignored, particularly as maternal mortality related to unsafe abortion declines.

The social and cultural sensitivity of abortion, its rootedness in individuals’ core values, and the absence of a strong international consensus on abortion policy all argue for starting with national level agenda setting processes and factors. Theories focused on extranational forces behind policy change can also miss how such efforts can backfire, as seen in the reactions of the Ethiopian government and NGOs to U.S. policy attempts (Mexico City policy/Global Gag Rule) to forestall abortion law reform. International (U.S.) pressure may even strengthen the hand of reformers by inoculating them against charges of being “too Western.” Supporters of abortion law reform elsewhere may be able to gain legitimacy by pointing to U.S. government and U.S. religious organizations’ interventions opposing abortion law reform. A final argument for the primacy of national actors and forces in Ethiopia’s reform that merits further investigation looks outside the country: other countries exposed to the same sets of international actors and influences related to abortion have not enacted the notable reform that Ethiopia did.

## Supplementary Information

**Additional file 1.** Interview guide.

## Data Availability

To protect study participants’ anonymity, and to adhere to the requirements of our Institutional Review Board, the transcripts generated and analyzed during the current study are not publicly available. However, they can be discussed with the corresponding author on reasonable request.

## Abbreviations

AWG: Advocacy Working Group
EOC: Ethiopian Orthodox Church
EPRDF: Ethiopian People’s Revolutionary Democratic Front
ESOG: Ethiopian Society of Obstetrician-Gynecologists
EWLA: Ethiopian Women Lawyers’ Association
MSIE: Marie Stopes International Ethiopia
SCIA: Supreme Council of Islamic Affairs
SSA: Sub-Saharan Africa
TPLF: Tigray People’s Liberation Front
UN: United Nations
UNFPA: United Nations Population Fund
U.S.: United States of America
WHO: World Health Organization

## Footnotes

1 However, the government’s orientation to religion has changed under the leadership of President Abiy Ahmed, a Protestant Pentecostal.
