# Supplementary material for: Agenda setting and socially contentious policies: Ethiopia’s 2005 reform of its law on abortion
Source: Reprod Health. 2022 Jun 13;19(Suppl 1):218. doi: 10.1186/s12978-021-01255-z (PMC9195348; doi:10.1186/s12978-021-01255-z)
Supplement: Supplementary file 1 — Additional file 1. Interview guide. [file 12978_2021_1255_MOESM1_ESM.docx]

## Agenda-setting and socially contentious policies: Ethiopia’s 2005 reform of its law on abortion

**Key Informant Interview Guide Questions**

*Before we start, I want to emphasize that your name and all the answers that you give during this interview will be kept strictly confidential. The only people who will see your name will be me and the professors supervising this research. As mentioned on the consent form, I will be recording the interview. If at any time you wish me to stop recording, I will do so. We will use a number instead of your name on your interview form and on the digital audio recording. Once we transcribe the digital recording, we will destroy all copies of the digital file.*

*I assume that your answers will be based on your experience with the field of reproductive health in Ethiopia -- but please do feel free to give broader answers based on your knowledge of the health sector and government in Ethiopia generally. There are, of course, no wrong answers to the questions I am about to ask you. I want most of all to learn about what you think about the questions, because you are the one with the knowledge and experience and relevant opinions.*

*Thank you for taking the time to participate in this interview and to share your knowledge, opinions and ideas.*

***Background***

1. **Could you start off by giving me a little background on yourself? What was your path to the position you now have?**

***Temporality; Sequence, Actors: what happened; who was involved***

*As you know, in 2005 as part of efforts to update Ethiopia’s penal code and have it reflect Ethiopia’s new constitution, the penal code regarding abortion were reformed to allow abortion in the cases of risks to health, minors, socioeconomic need, rape, and incest among other indications.*

1. **What is your understanding of how the 2005 penal code reform related to abortion took place? What were the key steps/stages in the reform process? (**How familiar are you with this reform?)
   1. **Why was the reform done as part of the penal code as opposed to as a separate, independent reform?**
2. Who were the key actors involved?
   1. **What parts of the Ethiopian national government were engaged in the policymaking in support of the penal code reform?** Which were the most influential (drivers)? Were there government officials who did not feel this reform was a priority? Why?
   2. **What were the organizations and sectors outside of government that were involved in penal code reform? I am asking about both supporters AND opponents. First, who were the supporters? Second, who were the opponents?**

*Now I have two questions about how the penal code reform was discussed (language, concepts, and reasoning used) and where it was discussed.*

1. **How did Government and NGO leaders talk about the 2005 penal code reform?** What reasons were given for and against it? What types of words and ideas did people use to describe the reform? (Slogans? Key words?)
2. **Where did most of the discussions about penal code reform take place?** How much was the penal code reform discussed in public? Why?
3. **Is there an organization or an individual without whom this policy reform would not have happened? If yes, who are they and what did they do?**
4. **What do you think were the most effective strategies used in support of penal code reform?** (by opponents?)

*Now I want to ask about whether people’s religious beliefs or whether religious leaders and institutions (such as the Patriarch or the Secretary General of the Islamic Affairs Council and priests or imams) had an effect on the penal code reform process.*

1. **What are the key religious beliefs in Ethiopia related to abortion? (among Christians, Muslims, traditional/animist beliefs)? What role, if any, do you think organized religion and people’s individual religious beliefs played in the penal code reform process?**

As you know, sometimes donors, with their funding, can try to influence a country’s health sector priorities – one example would be PEPFAR which seeks to influence policy on HIV/AIDS.

1. **Do you think donor agencies (UNFPA, WHO, USAID, private foundations) contributed at all to having the 2005 penal code reform take place (directly or indirectly), which ones?**
2. **How were international NGOs involved in the penal code reform? Were these iNGO staff members Ethiopian or were some non-Ethiopian?**

NGOs

1. **What were the top policy priorities of your organization in the 2003 - 2004 – 2005 period?** (Was penal code reform one of the top 3 priorities?)
2. **To what extent do you and your organization have connections with advocates in other SSA countries who do work to reform national abortion laws?**

***Summative Questions - #14 & 15 to be answered by everyone***

1. **If you had to choose the 1-2 most important factors explaining why the 2005 penal code reforms took place, what would they be?**
2. **Why do you think that reform in the penal code (in the law on abortion) did not happen before 2005? (**Prior to 2005, were there other times that reform in the penal code regarding abortion was actively considered?)
3. **How do you think implementation of the new law is going? Why?**

**Wrap-up Question**

Are there any other points related to policy making on maternal mortality and abortion in Ethiopia that you think that I should hear?
